# Regulation of protein homeostasis in neurodegenerative diseases: the role of coding and non-coding genes

**DOI:** 10.1007/s00018-015-1985-0

**Published:** 2015-07-21

**Authors:** Olga Sin, Ellen A. A. Nollen

**Affiliations:** European Research Institute for the Biology of Aging, University of Groningen, University Medical Centre Groningen, 9700 AD Groningen, The Netherlands; Graduate Program in Areas of Basic and Applied Biology, Abel Salazar Biomedical Sciences Institute, University of Porto, 4099-003 Porto, Portugal

**Keywords:** Protein homeostasis, Genetic modifiers, Non-coding RNA, Protein aggregation, Neurodegeneration, Alzheimer’s disease, Parkinson’s disease, Huntington’s disease, *C. elegans*, Proteotoxicity, Protein quality control, Proteostasis, tRNA, iPOD, JunQ, Aggresome, Chaperone, miRNA

## Abstract

Protein homeostasis is fundamental for cell function and survival, because proteins are involved in all aspects of cellular function, ranging from cell metabolism and cell division to the cell’s response to environmental challenges. Protein homeostasis is tightly regulated by the synthesis, folding, trafficking and clearance of proteins, all of which act in an orchestrated manner to ensure proteome stability. The protein quality control system is enhanced by stress response pathways, which take action whenever the proteome is challenged by environmental or physiological stress. Aging, however, damages the proteome, and such proteome damage is thought to be associated with aging-related diseases. In this review, we discuss the different cellular processes that define the protein quality control system and focus on their role in protein conformational diseases. We highlight the power of using small organisms to model neurodegenerative diseases and how these models can be exploited to discover genetic modulators of protein aggregation and toxicity. We also link findings from small model organisms to the situation in higher organisms and describe how some of the genetic modifiers discovered in organisms such as worms are functionally conserved throughout evolution. Finally, we demonstrate that the non-coding genome also plays a role in maintaining protein homeostasis. In all, this review highlights the importance of protein and RNA homeostasis in neurodegenerative diseases.

## Protein homeostasis

### Protein folding

**Fig. 1:**
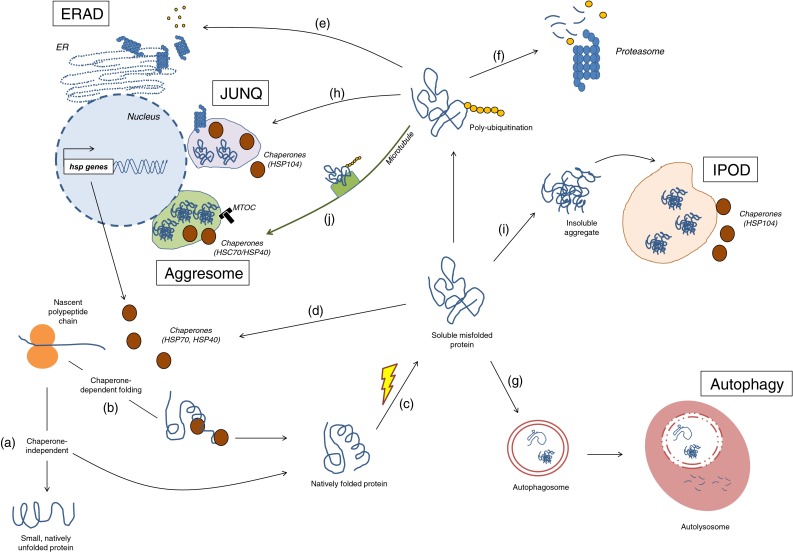
Quality control of cellular proteins. When a protein is synthesized, it can acquire its native state in a chaperone-independent (*a*) or dependent (*b*) manner. Upon environmental stress or mutations, the protein may either not acquire its native state or lose it, both leading to misfolding (*c*). Here, the misfolded protein can be refolded back to its functional conformation with the aid of chaperones (*d*); or sent to degradation via the ERAD (*e*), the ubiquitin–proteasome system (*f*) or autophagy (*g*). Alternatively, it can be redirected to the JUNQ for posterior refolding or degradation by the proteasome (*h*) or it can be permanently sequestered in the IPOD (*i*) or aggresome (*j*)

Maintaining a healthy proteome is important to ensure cell survival and function. The cell maintains a healthy proteome through a series of complex and tightly regulated surveillance systems (Fig. 1). These systems ensure that each protein is properly folded or assembled in a state that is required for it to perform its function in the cell.

After the synthesis of a nascent polypeptide chain, the protein’s amino acid sequence determines whether or not the protein becomes folded, and whether or not chaperone proteins are required for its folding (Fig. 1a, b). Some proteins are thought to exist in a predominantly “unfolded”, “disordered” or “intrinsically unstructured” state ([1], also reviewed in [2, 3]). Such proteins are typically involved in transcription, in signaling pathways and in protein networks ([4], also reviewed in [5, 6]). In mammals, about half of all possible proteins are predicted to have long disorganized regions and about 25 % are estimated as being intrinsically unstructured [2]. Other proteins have domains within their amino acid sequence that can fold spontaneously, whereas other large, multi-subunit proteins require molecular chaperones to assist in folding to their native state, as shown in in vitro studies [7–11].

The molecular chaperones that cooperate in the *de novo* folding or refolding process are subdivided into different classes, which include the Hsp70 system, the small chaperones, the chaperonins and the Hsp90 system [11–14]. In the case of *de novo* synthesis, chaperones protect the nascent polypeptide chain from aberrant contacts with other domains of the same proteins and from aggregation with other proteins (Fig. 1b) ([13, 14], also reviewed in [12, 15]). As a protein is synthesized, it is transiently unfolded and its hydrophobic regions are exposed. Hsp70 is able to recognize these regions and it binds to the protein substrate via its peptide-binding site in an ATP-dependent manner (reviewed in [12, 15, 16]). Hsp70 holds the substrate in an extended conformation, stabilizing it and preventing premature misfolding and aggregation. Next, the substrate can be transferred to another chaperone system, such as the chaperonins, where folding takes place and a three-dimensional structure is acquired (reviewed in [12, 16, 17]).

When misfolded proteins accumulate, unfolded protein responses can increase the levels of chaperones, which are then able to restore the proteins to their properly folded form (Fig. 1c, d, reviewed in [16, 18–21]). Such an accumulation of misfolded protein is just one of the types of stress that can trigger unfolded protein responses. Unfolded protein responses are mechanisms that are highly conserved from yeast to humans and that are induced upon environmental and physiological stress, such as thermal or oxidative stress (reviewed in [22–24]). In one of these pathways thought to respond to the accumulation misfolded proteins in the cytosol, heat shock factor 1 (HSF-1) acts as a master transcriptional regulator. HSF-1 is activated upon phosphorylation, after which it translocates from the cytosol to the nucleus to bind to the so-called heat shock elements, thereby upregulating the transcription of heat shock genes. These genes are then translated into proteins that assist in the refolding of misfolded proteins into functionally active proteins, in preventing unspecific interactions, or in mediating their degradation (Fig. 1d) (reviewed in [19, 22]).

Another strategy used by the cell to restore protein homeostasis is the unfolded protein response that is associated with the endoplasmic reticulum (ER) (Fig. 1e, also reviewed in [18, 25, 26]). The ER is the organelle where proteins enter the secretory pathway to acquire post-translational modifications, after which they are delivered to their corresponding organelle, fixed in the plasma membrane or shuttled outside of the cell to perform their function [27]. If misfolded proteins accumulate, the ER-associated degradation (ERAD) pathway is activated through signal transduction pathways that are mediated by three upstream effectors: inositol-requiring protein 1 (IRE1), activating transcription factor (ATF)-6 and PKR-like endoplasmic reticulum kinase (PERK).

IRE1, ATF-6 and PERK mediate three distinct pathways. Firstly, IRE1 is a transmembrane protein kinase that activates itself by auto-phosphorylation and mediates splicing of Hac1 in yeast and XBP-1 in eukaryotes [28–32]. IRE1 is known to promote the transcription of three groups of genes: stress-responsive genes including molecular chaperones and folding enzymes, genes involved in ERAD and genes involved in ER trafficking [33–35]. Secondly, ATF-6 is a transmembrane protein with a transcription factor domain (leucine zipper) that translocates from the ER lumen to the Golgi apparatus to be cleaved by proteases [36, 37]. This proteolysis releases the ATF-6 cytosolic fragment, which then enters the nucleus to induce the transcription of ER-resident chaperones and the transcription factor XBP-1, thereby increasing ER protein quality control capacity [29, 37–39]. Thirdly, PERK is a transmembrane kinase protein that phosphorylates the alpha-subunit of the eukaryotic translation initiation factor 2a (eIF2a), thus preventing the binding of the initiator tRNA(Met) to the ribosomal complex, necessary for translation initiation [40–42]. This results in an overall reduction in protein synthesis, thereby attenuating the accumulation of misfolded proteins at the ER.

### Protein degradation

If an aberrant protein cannot be folded back into its native state by the molecular chaperones, then it can be eliminated by two proteolytic systems, the proteasome and autophagy (Fig. 1f, g). In the degradation via the ERAD pathway, the ER cooperates tightly with the ubiquitin–proteasome system (UPS) to recognize, mark and traffic the misfolded proteins to the cytosol for degradation (Fig. 1e, reviewed in [18, 43–45]). The exact mechanisms that allow the cell to discriminate misfolded proteins from correctly folded proteins are not fully understood (reviewed in [44, 46, 47]). However, the current notion is that misfolded proteins can be recognized by molecular chaperones (the HSP70 family of proteins) and co-chaperones (the DnaJ/HSP40 family of proteins) [48–51].

An example that illustrates this recognition is the immunoglobulin binding protein (BiP), an HSP70 chaperone that recognizes and binds to the hydrophobic regions of misfolded proteins, thereby preventing their aggregation [49–53]. The binding of the ERAD substrate to BiP and its subsequent release depends on the conversion of ADP to ATP, a process regulated by ERdj proteins, which are part of the DnaJ/Hsp40 family of co-chaperones, and the nucleotide exchange factors GRP170 and BAP/Sil1 [48, 52]. These factors stimulate the ATPase activity of BiP and stabilize its binding to the misfolded protein [54–58]. The ERdj co-chaperones have also been shown to bind directly to unfolded proteins, thus maintaining them in a soluble state to be later recruited by BiP [48, 59]. After the misfolded protein has been identified, it is poly-ubiquitinated to be subsequently targeted for degradation [60–62].

Ubiquitination is a sequential three-step process that marks proteins destined for the proteasome (Fig. 1f). It starts with the activation of ubiquitin (a small 76 amino acid protein) by the activating enzyme E1, followed by binding of ubiquitin to the active site of the ubiquitin-carrier protein E2 and, finally, transfer of the ubiquitin molecule to the substrate in a reaction catalyzed by the ubiquitin protein ligase E3. At least four ubiquitin molecules must be bound to the ERAD substrate for it to be later recognized by the proteasomal machinery [63, 64]. Following this step, the misfolded proteins are delivered to the proteasome (a process called retrotranslocation) and the ubiquitin molecules are removed from the substrate prior to degradation by the deubiquitinating enzymes and recycled [65–67]. The proteasome is a barrel-shaped, multicatalytic proteinase where proteolysis occurs and proteins are cleaved into peptides 2–30 amino acid long [68].

The second proteolytic system, autophagy (“self-eating”), is a cellular degradation mechanism that eliminates cytosolic components, organelles and pathogens via lysosomes (Fig. 1g, [69–72]). It is the part of the cell that ensures protein and organelle turnover, where old cellular components are degraded and recycled molecules become available for cell metabolism [70, 71, 73]. For the purpose of this review, we discuss only the role of autophagy as a protein quality control system.

Autophagy can be classified into three categories: macroautophagy, microautophagy and chaperone-mediated autophagy (CMA). In macroautophagy, a newly formed double membrane vesicle engulfs the cytosolic material, forming the autophagosome. The autophagosome then fuses with an endosome or lysosome, giving rise to the autolysosome where degradation takes place through the action of hydrolytic enzymes (Fig. 1g) [71]. The double membrane that surrounds the autophagosome is derived from the ER, the mitochondria or the plasma membrane [74–78]. In yeast, autophagy is a multi-step process that requires at least 37 autophagy-related (ATG) genes [79–89]. The majority of the ATG genes have shown to be functionally conserved in mammals [90, 91]. In microautophagy, small molecules from the cytoplasm are internalized by the lysosome through invagination of its own membrane [70, 73]. In contrast to autophagy and CMA, much less is known about microautophagy [92].

CMA differs from the former two forms of autophagy in that it does not involve membrane reorganization. Instead, substrates with a KFERQ amino acid motif are recognized by an HSP70 cytosolic chaperone, Hsc70, that binds and delivers them to the CMA receptor at the lysosome [93–96]. Here, the substrate is unfolded before it is translocated into the lumen of the lysosome for degradation, which is assisted by Hsc73, an intralysosomal HSP70 chaperone [97, 98].

Crosstalk exists between the UPS and autophagy. Chronic low-level proteasomal inhibition is known to be sufficient to activate autophagy, and it has been suggested that ubiquitinated proteins may also be eliminated through this pathway [99–101]. It has also been proposed that macroautophagy may occur as a compensatory mechanism when either the UPS or CMA is impaired [102, 103].

### Protein compartmentalization

An alternative pathway for misfolded proteins is the sequestration into specialized protein quality control compartments where they can be either recovered or permanently sequestered (Fig. 1h, i, j) ([104–109], also reviewed in [110, 111]). Distinct quality control compartments harbor different species of misfolded proteins and are evolutionary conserved from yeast to mammals [105, 107–109, 112, 113]. Ubiquitinated misfolded cytosolic proteins are assigned to the juxtanuclear quality control compartment (JUNQ, Fig. 1h). These soluble, mobile misfolded proteins can subsequently be recovered by the molecular chaperone Hsp104 and either refolded back into functionally active proteins or degraded by the proteasomes localized nearby (Fig. 1h) [108, 112]. Non-ubiquitinated misfolded proteins—comprising amyloidogenic proteins—are redistributed to the insoluble protein deposit (IPOD, Fig. 1i). This compartment is localized at the cell periphery and is known to contain insoluble and immobile species, which are not recoverable and seem to remain terminally sequestered there (Fig. 1i) [108]. More recently, it has been proposed that there are no pre-existing compartments in the cell, and that soluble ubiquitinated misfolded proteins (but not the non-ubiquitinated amyloidogenic type) may coalesce and form transient structures termed ‘Q bodies’ that eventually mature into the JUNQ compartments [104].

Much research has focused on finding out whether the redistribution of misfolded proteins to these spatial cytosolic compartments is a random event or whether it depends on the concerted action of sorting factors. Evidence suggests that the latter is the case, and that sorting factors interact with chaperones to deliver misfolded proteins to each compartment [105]. For example, upon physiological stress, Btn2 (a Hook family protein involved in linking organelles to microtubules) was shown to associate either with the yeast small heat shock protein Hsp42 to assign misfolded proteins to the IPOD or with the chaperone Sis1 to guide misfolded proteins to the JUNQ [105, 107].

Another type of cytosolic compartment—the aggresome—is localized at the microtubule organizing center (MTOC) and is formed when the proteasome is unable to clear misfolded proteins properly (Fig. 1j) [114]. Aggresome formation is accompanied by redistribution of vimentin, an intermediary filament that acquires a cage-like structure in the aggresome. Ubiquitinated misfolded proteins depend on microtubules to be transported to the aggresome, this being done by the dynein/dynactin complex (Fig. 1j) [115]. Interestingly, the JUNQ shares several properties with the aggresome, including its perinuclear localization, and the presence of chaperones and ubiquitinated misfolded proteins [108, 114]. It has also recently been shown to functionally associate with the MTOC and vimentin [112]. Indeed, continuous accumulation of misfolded proteins in the JUNQ is thought to turn it in an aggresome over time [112].

Similar structures to aggresomes are the so-called aggresome-like induced structures (ALIS), which were originally discovered in dendritic cells but were later also found in other type of cells [109, 116]. The ALIS is a transient structure with peripheral and juxtanuclear localization. It is induced under a wide variety of stress conditions (e.g., heat shock, starvation, oxidative stress, inflammation) and clusters newly synthesized, ubiquitinated misfolded proteins [106, 109]. ALIS substrates can also be cleared by the proteasome and lysosome [106].

Cell division could be considered as yet another protein quality control system that sequesters misfolded, aggregated proteins (reviewed in [117, 118]). Studies in bacteria and yeast have shown that accumulation of protein aggregates reduces the fitness of these cells, a problem partially resolved by asymmetric division: these protein deposits are retained in the aging mother cell while the daughter cells are freed from damaged proteins, a process also known as replicative rejuvenation [119–123]. In budding yeast, it has been shown that misfolded proteins sorted either to the JUNQ or IPOD remain in the mother cell after asymmetric cell division, thus avoiding passage of these species onto the daughter cells [124]. Follow-up work from the same group extended this observation to mammalian cells, where the JUNQ (but not the IPOD) continues to be inherited asymmetrically, thereby always freeing one of the two daughter cells from proteotoxicity [112].

While much is now known about the sophisticated quality control mechanisms that the cell has evolved to ensure proper protein homeostasis, several questions remain to be answered. We know that the cell relies on the concerted action of chaperones to prevent an unfolded or misfolded protein interacting aberrantly with other proteins until it can be refolded back into its native state. In case this is not possible, the aberrant protein is sent to be degraded via the ubiquitin–proteasome system or by autophagy. However, it is still not known how the cell chooses one mechanism of degradation over the other or whether the two mechanisms occur simultaneously. Another unknown relates to protein compartmentalization—yet another strategy for putting away proteins that need to be degraded or permanently sequestered. It has not yet been established how the cell can differentiate between degradable and non-degradable proteins and shuttle them to different subcellular compartments. Finally, another important question is how protein quality control changes during aging. Aging itself may be the contributing factor for progressive deterioration of protein homeostasis, impairing the ability of the protein quality control system to handle the equilibrium between protein folding and degradation.

## Protein misfolding and aggregation in neurodegenerative diseases

The effects of progressive deterioration of protein homeostasis are thought to play a role in age-related neurodegenerative diseases. The presence of protein aggregates in the brain is namely a hallmark shared by several neurodegenerative diseases, including Parkinson’s (PD), Alzheimer’s (AD) and Huntington’s disease (HD) (reviewed in [125, 126]). In these diseases, it is not yet clear why proteins accumulate into aggregates and how this relates to pathogenesis.

Protein aggregation and its relationship to aging and neurodegeneration have also been widely studied in animal models. Evidence from several animal models suggests that, as the animal ages, the cell’s stress response systems become less efficient and less capable of maintaining a balanced proteome [127–133]. This could lead to the progressive accumulation of cytotoxic aggregation-prone disease proteins that cannot be cleared, ultimately resulting in toxicity and cell death [100, 134–137]. In the roundworm *Caenorhabditis elegans,* a model organism much used to study aging, protein aggregation has been shown to occur during aging and to affect the lifespan of the organism [138–140]. As previously discussed, when a protein misfolds it exposes its aggregation-prone domains to the cellular environment—domains that would otherwise be structurally concealed—thereby facilitating the likelihood of aberrant interactions with other proteins, potentially leading to proteotoxicity. Such proteotoxicity is proposed to play a role in protein conformational diseases in humans, including PD, AD and HD.

The type of aggregates that are formed varies for different neurodegenerative diseases. Frontotemporal lobar degeneration with fused in sarcoma is an example of a neurodegenerative disease that is characterized by the presence of amorphous, non-amyloidogenic aggregates ([141, 142], also reviewed in [143]). On the other hand, the common neuropathological feature of PD, AD and HD is the presence of an aggregation-prone disease protein that acquires amyloidogenic properties, causing it to form intracellular amyloid aggregates or extracellular amyloid plaques in the brains of patients (reviewed in [125, 126, 144]). The amyloids present in these neurodegenerative diseases can be distinguished from other amorphous, unstructured aggregates because they are organized, insoluble fibrils with a cross-beta structure and because they can be detected by specific amyloid-binding dyes, namely Congo red and thioflavin T (reviewed in [145, 146]). It is interesting to note that—despite their differences in amino acid sequence and function—several unrelated aggregation-prone disease proteins have one thing in common: in disease they are present as amyloid. This suggests that their ability to form amyloid is related to disease and that they may cause proteotoxicity in a similar manner.

*In vitro* studies have made clear that virtually any protein can form amyloid fibrils under certain conditions. Such conditions include low pH, high temperature and high pressure [147–154]. Native proteins are known to exist in equilibrium with their partially unfolded state, and when they are destabilized by certain conditions or mutations, the equilibrium shifts towards amyloid formation. Predicting aggregation-prone regions in proteins is now possible using bioinformatic tools. Examples of such tools are TANGO, which can specifically identify regions prone to form beta sheets, and Waltz, which can distinguish between amyloid sequences and amorphous beta-sheet aggregates [155, 156].

**Fig. 2:**
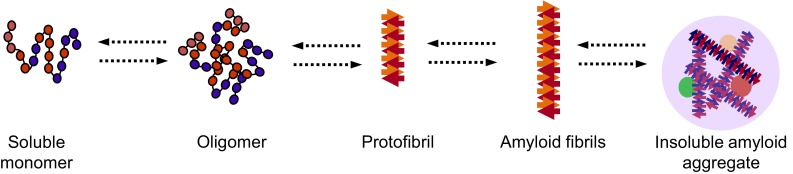
Proposed mechanism for amyloid formation. A protein loses its monomeric native state by conversion into an oligomer which can grow further into amyloidogenic fibrils and ultimately into insoluble amyloid aggregates

A proposed mechanism for amyloid formation is depicted in Fig. 2. Most of our understanding of this pathway has come not only from in vitro studies of aggregation-prone proteins, including amyloid-beta (seen in AD) and alpha-synuclein (seen in PD) but also from studies of globular proteins, including human lysozyme, superoxide dismutase 1, transthyretin and the acylphosphatase from the archaea *Sulfolobus solfataricus* (reviewed in [17, 125, 126, 146, 147]). One common step of amyloid formation appears to be the conversion of the monomeric, native state protein into an oligomeric intermediate state (Fig. 2). An oligomer is a small and transient cluster of protein molecules that has no fibrillar structure and is of low molecular weight [157–159]. These oligomers can then form protofibrils, which are fibrils 6 to 8 nm in diameter, about 200 nm in length and known to contain beta sheets detectable by Congo red and thioflavin T staining (Fig. 2) [160, 161]. Protofibrils can then convert into amyloid fibrils (Fig. 2) [160]. Of all these aggregation intermediates, it is currently thought that the early ones are cytotoxic and that aggregation may be a neuroprotective response to permanently sequester these intermediates, thereby preventing potentially toxic interactions with other proteins in the cellular milieu [162–165]. In support of this hypothesis, it has been shown that proteins rich in beta-sheet structures aggregate with newly synthesized proteins that have not yet become folded or with intrinsically unfolded proteins, thereby reducing the availability of these proteins to perform their normal function [135]. Further evidence demonstrating that oligomeric or protofibrillar forms of aggregation-prone disease proteins contribute to cell toxicity and death is reviewed elsewhere [144, 146, 166–168].

In a nutshell, the amyloid pathway has only just started to be described and it is not fully understood how protein aggregation correlates with disease. At the clinicopathological level, it is striking that there are individuals with high AD pathology (i.e., abundant amyloid deposits and neurofibrillary tangles) that yet do not display any cognitive impairment (reviewed in [169]). This fact makes it difficult to discern what are the boundaries between normal aging and disease. At the cellular and molecular level, what structural properties do aggregation-prone proteins acquire that make them toxic? This question is further complicated by the fact that aggregation-prone proteins such as amyloid-beta, huntingtin or alpha-synuclein do not share sequence, structure or function. A second question is that of how long neuronal cells can deal with these aggregation-prone proteins. And is their slow accumulation in the brain a reflection of an impaired protein quality control system? Finally, the majority of our knowledge about aggregation intermediates has come from in vitro studies. It remains to be shown whether oligomeric and fibrillar species exist in vivo and what their relevance to pathogenesis is.

## Genetic modifiers of proteotoxicity

### Genetic screens in small model organisms for protein aggregation in disease

The current understanding of how protein misfolding and aggregation contributes to neurodegeneration is far from complete. Molecular and cellular mechanisms that may regulate neurodegenerative disorders have been discovered in small organisms, the major ones being yeast (*Saccharomyces cerevisiae*), fly (*Drosophila melanogaster*) and nematode (*C. elegans*) (Table 1). In general, these small organisms are easy to grow and manipulate; their genomes are fully sequenced and accessible in public databases; and they provide information relatively quickly due to their short life cycle. Moreover, the availability of resources such as genome-wide mutant libraries (deletion, overexpression or RNAi-based) further adds to the attraction of using these organisms as powerful genetic tools. Indeed, the well-established models of several neurodegenerative diseases, including AD, PD and polyglutamine diseases, have now been generated in each of these small organisms [170]. Of note is that expression of an exogenous aggregation-prone protein typically exclusive to mammals can faithfully mimic some neuropathological features, namely the protein aggregation and toxicity phenotype seen in the diseased brain [170, 171]. And it is this that makes models in small organisms so attractive in the search for evolutionary conserved modifiers of proteotoxicity. These modifiers will provide insight into disease pathology and can be further explored as targets for therapy.

**Table 1:** Overview of a selection of genetic modifiers of protein aggregation and toxicity that are conserved between small model organisms, mammalian cells and animal models

Disease	Organism	Transgene	Tissue	Modifier	Mammalian ortholog	Cellular process	Transposed to	References
PD	*S. cerevisiae*	Alpha-synuclein	na	YPT1P	RAB1	ER-to-Golgi vesicular trafficking	*D. melanogaster, C. elegans* and rat midbrain primary neurons	[176]
	*S. cerevisiae*	Alpha-synuclein	na	YPK9	ATP13A	Manganese homeostasis	*C. elegans* and rat midbrain primary neurons	[177, 178]
	*C. elegans*	Alpha-synuclein	Body wall muscle and dopaminergic neurons	ATGR7	ATG7	Autophagy	Mouse	[181, 183]
AD	*C. elegans*	Amyloid-beta	Body wall muscle	DAF-2	IGFR1	Insulin/IGF–1 signaling pathway	Mouse	[195, 197]
	*S. cerevisiae*	Amyloid-beta	na	YAP1802	PICALM	Endocytosis	*C. elegans*, rat cortical neurons, human	[198]
	*D. melanogaster*	Tau	Eye	GLUT1	SLC2A14	Glucose metabolism	Human	[199]
PolyQ	*C. elegans*	Polyglutamine	Body wall muscle	MOAG-4	SERF1A and SERF2	Unknown	Human HEK293 and SK-N-SH cells	[203]
	*C. elegans*	Polyglutamine	Touch receptor neurons	662 modifiers	239 genes, of which 49 diferentially expressed in HD mouse brain	Diverse	CHL2 and R6/2 mouse models	[205]
	*C. elegans*	Polyglutamine	Body wall muscle	CCT	TRiC	*De novo* protein folding	*In vitro,* *S. cerevisiae*, mouse N2A neurons, human HeLa cells	[202, 206, 207]
	*S. cerevisiae*	*huntingtin* fragment with expanded polyglutamine	na	BNA4	KMO	Kynurenine pathway of tryptophan degradation	*D. melanogaster*, mouse	[208–210]

*PD* Parkinson’s disease, *PolyQ* polyglutamine diseases, *AD* Alzheimer’s disease, *na* not applicable

Finding modifiers of proteotoxicity in such models can be relatively quick: researchers can take advantage of high-throughput screening techniques using genome-wide overexpression, deletion, or RNAi libraries or using chemical mutagenesis. These resources are unbiased methods that can be used to screen for genes that—when mutated, overexpressed or suppressed—contribute to an increase or decrease of protein aggregation and toxicity. Some of the hits that result from these screens may very well be genes that have already been associated with disease in humans. On the other hand, it is also a way of identifying previously unknown regulators of proteotoxicity—such findings may provide mechanistic insights into that particular disease. It should be noted, however, that genes shown to strongly suppress or enhance aggregation in one model do not always have a similar effect in other models, possibly due to the inherent differences between species or between the methods employed. Nevertheless, functionally conserved genetic modifiers of aggregation and toxicity have been identified across species.

In the end, to establish the value of genes discovered to be involved in aggregation and toxicity in small organisms, the results will have to be reproduced in human neurons and in mammalian animal models. If the function of modifiers of proteotoxicity identified in small organisms is evolutionarily conserved, their mammalian counterparts may become therapeutic targets worthy of future pharmacological investigation (Table 1). At the same time, small model organisms provide a simple platform that can be used not only to understand the basic mechanisms underlying the causal gene of disease but also as a pharmacological screening tool. Below we describe examples of genetic modifiers that have been studied in different model organisms for PD, AD and polyglutamine diseases.

### Parkinson’s disease models

Alpha-synuclein is the major constituent of the protein aggregates found in the brains of PD patients, which are also known as Lewy bodies [172]. It is a 140-amino acid protein that is mostly expressed in the brain and is thought to have a function at the synapse (reviewed in [126, 173]).

The aggregation phenotype is successfully recapitulated in the budding yeast *S. cerevisiae*, where heterologous expression of alpha-synuclein induces toxicity in a concentration-dependent manner and is associated with the formation of cytoplasmic protein aggregates similarly to those observed in the human brain [174]. The characteristics that make yeast a powerful genetic tool for studying neurodegenerative disorders are reviewed elsewhere [171, 175].

In yeast, Cooper et al. demonstrated that overexpression and subsequent accumulation of alpha-synuclein impairs vesicle transport from the ER to the Golgi (Table 1) [176]. In the same study, a genome-wide overexpression screen identified the small GTPase Ypt1 as a modifier of alpha-synuclein toxicity. Overexpression of Ypt1p was sufficient to prevent alpha-synuclein toxicity, by enabling forward trafficking from the ER to the Golgi. This observation was further extended to *Drosophila* and *C. elegans* models of PD as well as in rat midbrain primary neurons, where Rab1—the functionally conserved ortholog of Ypt1p—suppressed dopaminergic neuron loss (Table 1) [176].

Another modifier of proteotoxicity identified from the same original yeast screen was YPK9, an ortholog of the human lysosomal P-type ATPase ATP13A2 (also known as PARK9), an enzyme known to be associated with early onset parkinsonism (Table 1). YPK9 overexpression prevented alpha-synuclein-induced toxicity by reducing intracellular aggregation and restoring alpha-synuclein localization to the plasma membrane [177]. The same study showed that the *C. elegans* ortholog CATP-6 partially prevented dopaminergic neuron loss, and that knockdown of CATP-6 increased alpha-synuclein misfolding in an age-dependent manner. Finally, in rat primary neuron cultures transduced with a lentivirus carrying the familial alpha-synuclein A53T mutation, heterologous expression of human ATP13A2 prevented neuronal loss (specifically dopaminergic neurons). Notably, this study was the first to show a link between environmental and genetic causes of PD, since YPK9 protected against manganese toxicity in yeast, a heavy metal thought to be risk factor for PD. Indeed, YPK9 was later shown to regulate manganese tolerance via diverse cellular processes, such as vesicle transport, vacuolar organization and chromatin remodeling in yeast (Table 1) [178].

The important role of vesicle-mediated transport in alpha-synuclein toxicity has also been demonstrated by other studies [179, 180]. In a screen performed by Kuwahara et al., the authors discovered ten neuroprotective genes, four of which were involved in endocytosis. Knockdown of two of these genes (*apa*-*2* and *aps*-*2,* encoding two different subunits of the AP-2 adaptor protein which mediates clathrin-dependent endocytosis) revealed that deficiencies at synaptic vesicles led to alpha-synuclein neurotoxicity [179].

Several modifiers of proteotoxicity have also been identified using RNAi screens in *C. elegans* models of PD [179–181]. Follow-up on this work has revealed *tdo*-*2* as a general regulator of proteotoxicity and lifespan [182].

Genetic screens not only help us to identify novel modifiers of proteotoxicity, but they can also be useful for rediscovering genes that were previously known to be associated with disease. Such an example comes from work by Hamamichi et al., where an RNAi screen identified the autophagy-related gene *Atgr7* as protecting against alpha-synuclein-induced toxicity in *C. elegans* dopaminergic neurons [181]. The mammalian ortholog of *Atgr7* has previously been implicated in neurodegeneration in mice, where it was found to cause axonal degeneration and dystrophy when ablated, thereby highlighting the importance of neuronal autophagy in preventing degeneration (Table 1) [183].

### Alzheimer’s disease models

The brains of patients with AD are characterized by the presence of amyloid plaques and neurofibrillary tangles, which develop as a result of an accumulation of extracellular deposition of two different proteins: amyloid-beta in the plaques and intracellular hyperphosphorylated tau in the tangles (reviewed in [184, 185]). The disease can be caused by a mutation in the gene for amyloid precursor protein (APP), or in presenilin 1 or presenilin 2, all of which alter amyloid production (reviewed in [184, 186]).

*C. elegans* has been a fundamental tool for dissecting the pathways that link lifespan to AD (Table 1). Specifically, one of the major pathways that regulates lifespan is the insulin/IGF-1 signaling (IIS) pathway—a pathway that has been validated in nematodes, flies and mice and strongly implicated in humans [187–193]. In one of the models that recapitulates AD, *C. elegans* expresses a human amyloid-beta protein fragment (peptide 3–42) in the body wall muscle and progressive paralysis is used as readout for amyloid-beta toxicity [194]. In this model, knockdown of the insulin/IGF-1 receptor DAF-2 not only significantly extended lifespan but also prevented amyloid-beta toxicity by delaying the onset of paralysis, identifying a link between the mechanisms of aging and proteotoxicity [195]. Modulation of lifespan by DAF-2 was also found to be highly dependent on HSF-1 and DAF-16, two transcription factors known to drive the expression of longevity genes [196]. Curiously, while both blocked proteotoxicity, they did so through opposing effects: while HFS-1 promoted disaggregation, DAF-16 pushed aggregation forward, possibly as a means of sequestering the amyloidogenic protein from the cellular milieu [195].

The observation that inhibition of the IIS pathway protects against proteotoxicity was further confirmed in an AD mouse model with haploinsufficiency of IGFR-1, the mammalian ortholog of DAF-2 (Table 1) [197]. Here, reducing only half the expression of IGFR-1 (and thereby the IIS pathway) was sufficient to prevent amyloid-beta toxicity, namely by reducing inflammation and neuron loss. The AD mice with reduced IGFR-1 also performed better in memory and learning tasks than their age-matched AD controls did and this was found to be correlated with the formation of densely packed aggregates in the brain. This supports the idea that aggregation is a protective mechanism to permanently sequester smaller, soluble oligomeric amyloid-beta species that are proteotoxic.

The importance of modeling neurodegenerative diseases in small organisms has been further reinforced by Treusch et al., who have identified modifiers of amyloid-beta toxicity that are conserved from yeast to humans (Table 1) [198]. Taking advantage of a yeast model of AD, they performed an unbiased genetic screen for modifiers of amyloid-beta toxicity. Of the identified modifiers, six were found to be risk factors for AD in humans—either validated or potential—that had been previously identified from family-based genome-wide association studies (GWAS). These modifiers were specific to amyloid-beta, in that in yeast they did not prevent toxicity induced by another aggregation-prone protein, alpha-synuclein. Another modifier of amyloid-beta toxicity identified by Treusch et al. is YAP1802, a suppressor of amyloid-beta proteotoxicity that is involved in clathrin-mediated endocytosis. Its human homolog PICALM is also involved in endocytosis and has been validated as a high-risk factor for AD (Table 1). YAP1802 prevents amyloid-beta toxicity in yeast and the human homolog PICALM prevents amyloid-beta toxicity in rat cortical neurons. Notably, this study identifies a causal gene for susceptibility to AD and proposes defective endocytosis as a contributing factor in AD pathology, with a possible role for PICALM.

In another independent study, GWAS data for AD were combined with a functional screen in *Drosophila* (Table 1) [199]. From a set of GWAS variants obtained from patients with AD, Shulman et al. found 19 evolutionarily conserved orthologs in the fly that either enhanced or suppressed neurotoxicity associated with tau. Six of these interacted with tau in vivo, including the glucose transporter GLUT1, found to be functionally conserved in the human ortholog SLC2A14, further supporting a role for this risk factor as a disease modifying factor (Table 1) [199].

### Polyglutamine disease models

In addition to models for PD and AD, there are several other models for aggregation-prone proteins, which include those for human polyglutamine diseases such as Huntington’s disease. In polyglutamine diseases, trinucleotide repeats cause expanded tracts of the amino acid glutamine in the encoded protein. In one *C. elegans* model, the animals express expanded glutamine repeats fused to a fluorescent protein in the body wall muscle. Expression of 35–40 glutamines is sufficient to cause aggregation, which increases with aging and is correlated with toxicity [200]. This model has been used in at least two genome-wide RNAi screens performed to search for suppressors and enhancers of proteotoxicity [201, 202]. These screens identified genes involved in RNA metabolism, as well as in protein synthesis, folding, trafficking and degradation as polyglutamine modifiers. In a subsequent screen to look for more modifiers, it was found that polyglutamine aggregation is not always coupled with proteotoxicity [201].

In an EMS screen to find genes that drive aggregation, van Ham et al. identified MOAG-4 (modifier of aggregation) as an aggregation-promoting factor in disease models expressing polyglutamine, alpha-synuclein and amyloid-beta, establishing MOAG-4 as a general regulator of proteotoxicity (Table 1) [203]. MOAG-4 is thought to be active during the early steps of the aggregation process, where it drives the formation of compact aggregation intermediates [203]. MOAG-4 is functionally conserved in two human orthologs, SERF1A and SERF2, which have the same aggregation-promoting function in human cell-based models of polyglutamine diseases (Table 1) [203]. Recent insights into the function of one of these proteins, SERF1A, suggest that it acts as an amyloid-promoting factor [204]. In this study, SERF1A recognized a broad range of aggregation-prone proteins (alpha-synuclein, huntingtin, amyloid-beta, prion protein) and mediated their conversion into amyloid in vitro [204]. It was further demonstrated that, to do this, SERF1A interacted directly with the monomeric form of the protein to seed amyloid growth, therefore supporting the hypothesis that MOAG-4/SERF1A acts on the early intermediates of the amyloid pathway [204]. SERF1A did not promote aggregation of non-amyloidogenic proteins.

An RNAi screen performed by Lejeune et al. identified 662 modifiers that regulate polyglutamine-induced proteotoxicity in *C. elegans* touch receptor neurons, 49 of which were found to be differentially expressed in two mouse models of HD (Table 1) [205].

Another protein originally identified as a suppressor of polyglutamine aggregation in a *C. elegans* model is the chaperonin CCT [202]. It is composed of eight subunits and, together with HSP70, is involved in *de novo* folding of newly synthesized proteins [12]. Its ortholog, TRiC (also known as TCP), was shown to cooperate with HSP70 to prevent proteotoxicity by promoting the formation of non-toxic, soluble polyglutamine oligomers in a yeast model [206]. TRiC also modulated proteotoxicity in mouse and human cell models (Table 1) [207]. The subunit CCT1 was also shown to physically interact with polyglutamine to suppress aggregation in vitro, supporting the hypothesis that TRiC binds to polyglutamine to prevent it from acquiring a potentially toxic conformation [207].

Finally, a modifier identified in yeast is the kynurenine 3-monooxygenase BNA4, whose deletion prevented proteotoxicity induced by mutant huntingtin [208]. Follow-up work showed that genetic ablation or pharmacological inhibition of the ortholog KMO prevented toxicity in a fly and mouse model for HD (Table 1) [209, 210].

In summary, small model organisms including yeast, flies and nematodes are powerful tools for identifying genes involved in protein aggregation and toxicity. Several examples where small animal organisms complement findings from human cell models or mouse models further validate the importance of using these small animal models.

## Non-coding RNA in neurodegeneration

When the Human Genome Project started in 1990, it was estimated that 30,000–40,000 protein coding genes would be found in the human genome [211]. When the project was completed in 2001, researchers were surprised to find far fewer protein coding genes than expected, namely 21,000, representing only about 2 % of the total genome—with the remaining 98 % being considered as “junk DNA” [212, 213]. However, it soon became clear that this “junk DNA” actually contained regulatory elements such as non-coding RNA (ncRNA), transcription factor binding sites or certain chromatin structures that govern gene expression. These conserved functional elements in the human genome were subsequently comprehensively identified and characterized [214]. Within these conserved functional elements, many classes of ncRNA were identified and the list has been growing ever since ([215, 216], also reviewed in [217]). Indeed, the number of ncRNA transcripts is far greater than those coding for proteins and the list of all existing ncRNAs is not yet complete [218]. What we do know is that there are different classes of ncRNA with essential functions in gene transcription, RNA processing and translation, a selection of which is presented in Table 2 (a more complete list can be found in [217]). Indeed, impaired RNA metabolism has been correlated with several neurodegenerative diseases. For example, abnormal repeat expansions in the non-coding regions of disease-related genes induce toxic gain-of-function of RNA in myotonic dystrophy, amyotrophic lateral sclerosis, and frontotemporal dementia [219, 220]. For the purpose of this review, we focus on a few examples of ncRNAs that have been directly implicated in neurological or neurodegenerative diseases (Table 2).

**Table 2:** Classes of RNAs and their localization, size and function

Class	Localization	Transcribed by	Function	Size	Disease
Messenger RNA (mRNA)	Nucleus and cytoplasm	Pol II	Gene expression into protein	2–5 kb	na
Ribosomal RNA (rRNA)	Cytoplasm	Pol I	Translation, is part of the small or large subunit of the ribosome	70S (prokaryotic) or 80S (eukaryotic)	Unknown
Micro RNA (miRNA)	Nucleus and cytoplasm	Pol II or Pol III	Inhibition of translation or degradation of mRNA	21–23 bp	AD, PD, HD
Transfer RNA (tRNA)	Cytoplasm	Pol II or Pol III	Translation, brings cognate amino acid of mRNA to nascent polypeptide chain	70–90 bp	PHC-2, PHC-4, neurodevelopmental and neurological disease
Long non-coding RNA (lncRNA)	Nucleus and cytoplasm	Pol II or Pol III	Diverse regulatory roles of other RNAs and proteins, chromatin remodeling	>200 bp	AD, SCA7
Small nuclear RNA (snRNA)	Nucleus	Pol II or Pol III	Splicing of pre-mRNA	100–300 bp	SMA

*AD* Alzheimer’s disease, *PD* Parkinson’s disease, *HD* Huntington’s disease, *PHC* pontocerebellar hypoplasia, *SCA* spinocerebellar ataxia, *SMA* spinal muscular atrophy, *na* not applicable

### microRNAs

Over the past few years, it has become evident that ncRNAs are key players in the development and maintenance of the nervous system. Of all classes of ncRNAs identified so far, microRNAs (miRNAs) are those that have been most extensively studied and documented. The function of miRNAs is to bind to the 3′-untranslated region (3′ UTR) of messenger RNA and inhibit its translation or target it for degradation (Table 2) [221]. *In situ* hybridization studies in mouse and zebrafish have revealed miRNA to be expressed throughout the brain; these studies have also demonstrated that miRNA expression is spatiotemporally controlled, supporting a biological function for miRNAs in the central nervous system [222–224]. Indeed, several hundreds of miRNAs are involved in brain development [225–229]. miRNAs play a role in virtually every aspect of brain function including neurogenesis, neural differentiation and maintenance, and synaptic plasticity, all of which are described extensively elsewhere [230–232].

miRNAs have also been associated with various aspects of aging and neurodegenerative diseases (Table 2) [233–237]. For example, Northern blotting experiments in the hippocampi of fetuses, adults and AD patients have shown that miRNA expression changes during development and during aging [237]. In these experiments, miR-9 and miR-128 were upregulated in the AD hippocampus relative to age-matched controls, hinting that these miRNAs may be regulating the expression of genes required for pathogenesis. At least two other human studies have shown an association between miRNAs and the beta-site APP cleaving enzyme 1 (BACE-1), which is responsible for cleaving APP into the amyloid-beta 1-42 toxic species [235, 236]. In these studies, the expression of miR-107, miR-29a and miR-29b-1 was decreased in the AD brain while expression of BACE-1 was increased (Fig. 4a) [235, 236]. Since these miRNAs target the 3′ UTR of BACE-1, it follows that miRNAs can reduce BACE-1 mRNA levels and, therefore, amyloid-beta 1-42 generation in the brain, an effect that is lost in the diseased brain due to the reduced expression of these miRNAs (Fig. 4a) [235, 236]. In a mouse model for AD, miR-34a is thought to inhibit *bcl*-*2*, an anti-apoptotic gene that prevents cell death provoked by amyloidogenic species (Fig. 4b) [234]. Additionally, miR-124 has been found to regulate APP alternative splicing in neurons [233].

In PD, downregulation of the miR-34b/c cluster is correlated with downregulation of DJ-1 and Parkin, two genes implicated in the pathogenesis of PD, although a causal link has yet to be determined [238]. It has recently been shown in a cell model that this same cluster directly represses alpha-synuclein mRNA levels and consequently aggregate formation, establishing that miRNAs can have a direct effect on the expression of an aggregation-prone protein [239].

Several miRNAs have also been found to be dysregulated in polyglutamine diseases (Table 2) [240–244]. In HD, REST is a transcription factor that negatively regulates neuronal gene expression and has been found to repress brain-specific miRNAs in mouse and human brains [241, 242]. Two of these miRNAs, miR-9 and miR-9*, have been identified as targeting the REST complex in a negative feedback loop [244]. In a cell model of spinocerebellar ataxia type 1, miR-19, miR-101 and miR-130 cooperatively regulate ataxin-1 expression levels by binding to its 3′ UTR [243]. Inhibition of these miRNAs leads to ataxin-1 accumulation in cells and subsequent cell death [243].

### tRNAs

Transfer RNAs are essential for mRNA translation into a protein, as they are responsible for transporting the cognate amino acid to the nascent polypeptide chain (Table 2) [245, 246]. Due to the degeneracy of the genetic code, there can be up to five tRNAs per amino acid—termed isoacceptors—that have distinct anticodons for recognizing the same amino acid [245, 246]. On the other hand, tRNAs that share the same anticodon but have distinct body sequences are termed isodecoders, and their number vary greatly [246].

**Fig. 3:**
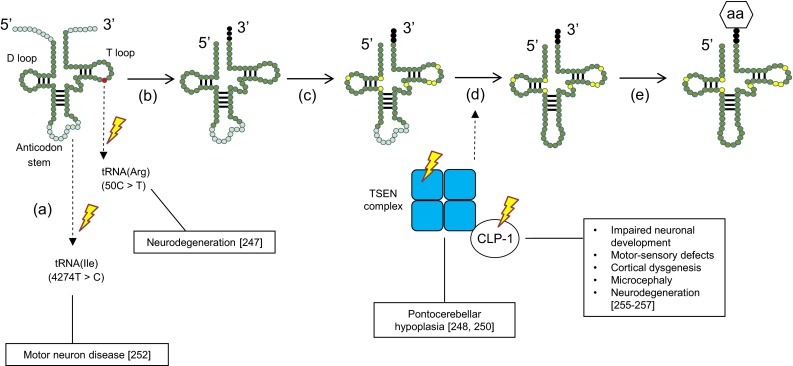
Mutations in the tRNA biosynthesis pathway that lead to neurodegeneration. The point mutation (50C>T) in the T loop of one tRNA isoacceptor for arginine (Arg) provokes neurodegeneration. Another described point mutation (4274T>C) in the mitochondrial tRNA for isoleucine (Ile) has also been associated with motor neuron disease (*a*). Following transcription, the 5′ leader sequence of the pre-tRNA is removed by RNAseP, the 3′ end is processed by RNAse Z and the trinucleotide CCA is added to the 3′ end by a nucleotidyl transferase (*b*). Different bases of the RNA transcript can undergo chemical modifications (*c*). The introns of the pre-tRNA are spliced out by a tRNA splicing endonuclease (TSEN). Mutations in these enzymes have been associated with pontocerebellar hypoplasia (PHC) and mutations in their co-factor CLP-1 with motor neuron loss (*d*). Finally, the mature tRNA is loaded with an amino acid (aa) via tRNA synthetases (*e*)

Growing evidence suggests that mutations in individual tRNAs—or in the enzymes involved in their biosynthesis—are a contributing factor in neurodegeneration (Fig. 3) [247–252]. For example, a point mutation (4274T>C) in the mitochondrial tRNA for isoleucine was identified in a patient suffering from motor neuron disease, although the mechanism by which this mutation might lead to disease is unknown (Fig. 3a) [252]. In a recent study, loss of function of one of the brain-specific tRNA isodecoders for arginine was found to be correlated with neurodegeneration in mice (Fig. 3a) [247]. Specifically, a point mutation (50C>T) in the T loop of the arginine tRNA provoked ribosome stalling, which is normally offset by GTPBP2. However, simultaneous impairment of GTPBP2 in these mice disabled its function as a so-called rescue factor, subsequently resulting in neurodegeneration [247].

Other impairments in the tRNAs biosynthesis pathway are seen in pontocerebellar hypoplasia (PHC). PHC is an autosomal recessive neurodegenerative disorder that has six subtypes (PHC1-6) and is generally characterized by hypoplasia and atrophy of the cerebellum and pons [253]. PHC2 and PHC4 arise from impaired tRNA splicing endonuclease (TSEN) activity. TSEN is composed of two catalytic subunits (TSEN 2 and TSEN34) and two non-catalytic subunits (TSEN54 and TSEN15) (Fig. 3c) [250, 254]. It is thought that mutations in both catalytic subunits and in TSEN54 may prevent proper complex formation, leading to misplicing of premature tRNAs (pre-tRNAs) into their mature form, thereby unbalancing the tRNA repertoire for protein synthesis [248, 250]. PHC6 results from a mutation in the intronic region of the mitochondrial pre-tRNA synthetase gene for arginine [251].

Finally, CLP-1 is a mammalian kinase that cooperates with the TSEN complex to remove the intronic loop of pre-tRNAs (Fig. 3c) [255]. Loss of CLP-1 results in severe impairment of spinal motor neurons in mice, ultimately leading to respiratory failure [255]. CLP-1 mutations in affected patients have been correlated with neurodevelopment and neurological symptoms in both the central and peripheral nervous system [256, 257].

In summary, these studies demonstrate a crucial role for tRNAs in neuronal function, as either mutations in their transcript or defective post-transcriptional modifications can affect their proper processing and function, ultimately leading to neurodegeneration.

### Other ncRNAs

**Fig. 4:**
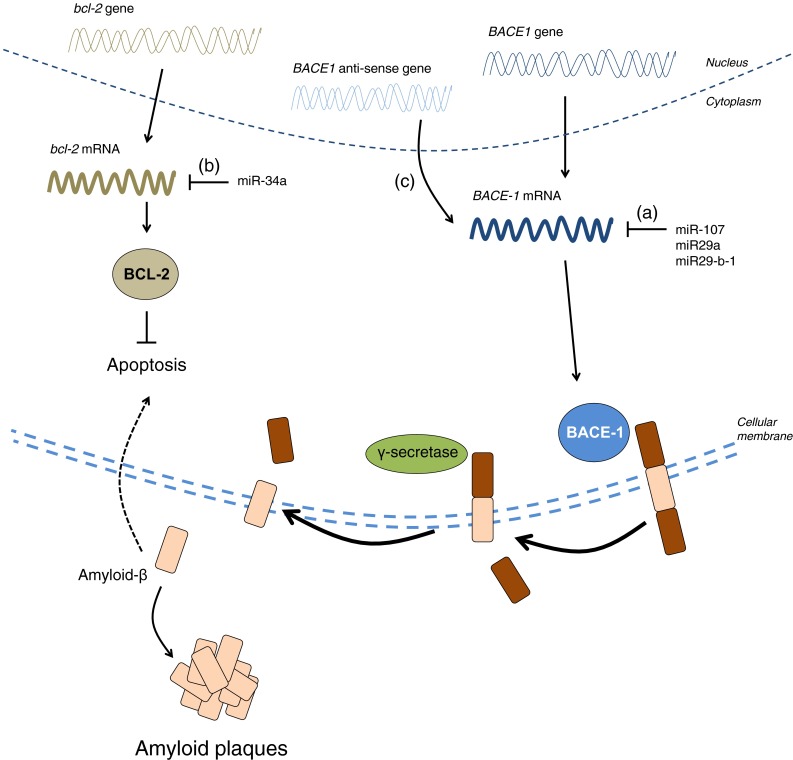
Impaired BACE-1 regulation contributes to AD. miR-107, miR-29a and miR29-b-1 were shown to be decreased in the brain of AD patients while BACE-1 mRNA and protein levels were elevated (*a*). In an AD mouse model, elevated levels of miR-34a negatively correlate with BCL-2 protein levels, which normally prevent apoptosis induced by amyloid-beta (*b*). BACE-1 anti-sense transcripts were reported to be upregulated in the brains of AD patients. BACE-1 anti-sense transcripts stabilizes BACE-1 mRNA, thereby facilitating its expression, which ultimately results in the generation of more amyloid-beta (*c*)

The other non-coding RNAs shown in Table 2 have been less well studied but are nevertheless worthy of mention. Long non-coding RNAs (lncRNAs) are more than 200 nucleotides long and are mostly expressed in the nervous system (Table 2) ([223], also reviewed in [258]). Three lncRNAs have been suggested to be involved in neurodegenerative diseases. Firstly, BACE-1 anti-sense transcript is an lncRNA that competes with miR-485-5p for binding to the BACE-1 mRNA to stabilize it (Fig. 4c) [259]. In AD, the levels of BACE-1 anti-sense transcript are elevated, thereby stabilizing BACE-1 mRNA and enhancing its expression, which further promotes the generation of toxic amyloid-beta 1–42 (Fig. 4c) [259]. Secondly, in spinocerebellar ataxia type 7 (SCA7), lncSCA-7 crosstalks with miR-124 to regulate transcript levels of *atxn7* [260]. Thirdly, Abhd11os is an lncRNA that has been shown to be neuroprotective against mutant *huntingtin* in two mouse models for HD, although the exact mechanism of how this occurs remains to be determined [261].

Small nuclear RNAs (snRNAs) exist as small nuclear ribonucleoproteins (snRNPs) and are major components of the pre-mRNA splicing machinery (Table 2) [262, 263]. The survival motor neuron protein (SMN) is directly involved in the generation of snRNPs [264]. In a mouse model of spinal muscular atrophy, SMN deficiency affects the snRNA pool in a tissue-specific manner, ultimately leading to pre-mRNA splicing defects in a diverse range of genes [264]. Further evidence for the involvement of snRNAs in neurodegeneration comes from work by Jia et al., who revealed that a mutation in a U2 snRNA gene impairs alternative splicing of pre-mRNA which is directly responsible for neuron loss in the cerebellum and hippocampus of mice [265].

Neurodegeneration is clearly not exclusively caused by imbalances in protein coding genes—it can also arise from dysregulation of ncRNAs. Over the past two decades, we have begun to understand that ncRNAs are not just “transcriptional noise” and have started to define their role in the CNS and in neurodegeneration. Several reports have shown that different classes of ncRNAs influence the expression levels of the disease protein and that each class of ncRNA does so either by affecting the protein post-transcriptionally or through crosstalk with other classes of ncRNAs (miRNAs, lncRNAs). Maintaining a proper environment for protein synthesis is crucial to ensure that each mRNA molecule is effectively spliced and translated into a protein (through tRNAs and snRNAs). To establish the causal relationships between changes in ncRNAs and disease phenotypes, the targets of these ncRNAs need to be uncovered. Understanding the role of ncRNAs will provide insight into the mechanisms of neurodegenerative diseases, which enables the identification of targets for therapeutic interventions.
